# Modes of transport in the Northeast Corridor: Dataset

**DOI:** 10.1016/j.dib.2019.104977

**Published:** 2019-12-12

**Authors:** Ignacio Escañuela Romana

**Affiliations:** Loyola University, Andalusia, Spain

**Keywords:** Demand, Maximisation, Elasticity, Transport, Megaregion

## Abstract

These data support the research article: ‘The elasticities of passenger transport demand in the Northeast Corridor’, Escanuela Romana, I. (2019) [1]. The necessary data were collected in order to be able to estimate a demand model for the different modes of transport between cities in the Northeast Corridor (NEC) of the United States. The data set includes the number of passengers, transport prices, its share within the budget of consumer expenses, for each one of the relevant passenger transportation modes: train, aeroplane, car and coach. The lack of official statistics on the number of passengers and road transport prices is confronted by reconstructing the series from the NEC freeway traffic meters. Such series shall, therefore allow us to estimate a multi-equational demand model in which the conditions of the rational consumer may be added and tested. Without this knowledge, it would not be possible to understand the elasticities and consider the most suitable maximising business strategies and public policies for the wellbeing of consumers.

**Table:** Specifications Table

Subject	Economics and Econometrics
Specific subject area	Transportation. Megaregions. Applied microeconomic theory.
Type of data	Table.
How data were acquired	Public repositories. The data were subsequently incorporated into an Excel spreadsheet (Microsoft) and the Rotterdam demand model was estimated in R (R: A Language and Environment for Statistical Computing, R Core Team, R Foundation for Statistical Computing, R version 3.5.2, 2018-12-20)*.*
Data format	Raw
Parameters for data collection	Set by public agencies. Data have been selected in order to be able to estimate a multimodal Rotterdam demand model and to cover an homogeneous period.
Description of data collection	From public agencies. Data were found in public repositories. The methodology used by the agencies is detailed in their repositories. Table 1100 (BLS), in relation to annual data series of the years 2003–2011, has been sent a prior request. The dataset includes: Number of passengers, prices and budget shares.
Data source location	Public and detailed repositories (these databases were accessed online from Seville, Spain).
Data accessibility	With the article.
Related research article	Escañuela Romana Ignacio, The elasticities of passenger transport demand in the Northeast Corridor, Research in Transportation Economics, 2019, 100759, ISSN 0739-8859, https://doi.org/10.1016/j.retrec.2019.100759. (http://www.sciencedirect.com/science/article/pii/S0739885919302719) [1]

**Table:** 

**Value of the Data** • First, these data series allow us to perform estimations concerning offer and demand of multimodal passenger transport in the NEC. • Second, these data are useful for the econometric estimation of any model referring to multimodal passenger transport in the NEC. • Third, these data and the model that they support propose an econometric focus in order to achieve an understanding of the demand and the offer, in the megaregions and, in general, on any transport route. • Fourth, these data series suggest how to improve the data available to the investigation in order to successfully advance in our knowledge of the transport mode and behaviour of the transport givers and demanders. • Fifth, the available data and the possible data and model extensions offer a procedure for businesses and public administrations in order to assess the effect of maximising business strategies as well as public policies. Understanding the preferences of the rational transport consumer would allow us to establish quantitative criteria for measuring gains and losses for the consumer's well-being.

## 1 Data

The data can be found in the attached calculation page: Elasticities_NEC_IER_Rev.xslx.

The data refer to the different modes of passenger transport in the NEC. They are annual data, from the 2003–2016 period. The variables are: number of passengers, average prices and average share of the consumers' budget. The fundamental characteristics of the transport infrastructures were not modified in said period, and therefore we have an almost homogeneous data series.

First, there is data on railway passenger transport in the NEC. This covers two Amtrak services: Acela and Northeast Regional, that primarily connects Boston, Providence, New York, Philadelphia, Baltimore and Washington DC. The data include the total number of annual passengers and the average annual prices of the service, weighted by the number of passengers. Source: [2,3].

Second, passenger air transport. The routes are: New York-Washington DC, Boston-New York, Boston-Philadelphia and Boston-Washington DC. The data include the annual number of passengers and the average prices per route. From there we can calculate the annual passengers and the average prices weighted by the number of passengers using this mode of transport. Source: Bureau of Transportation Statistics [13]. Method: Random monthly surveys on 10% of all tickets sold in the United States, U.S. DB1A data, U.S. domestic.

Third, data on car and coach transport. In order to calculate the number of passengers who use this transport, the calculation is based on the data available regarding the number of vehicles per freeway. The data are selected based on annual vehicle miles travelled (AVMT) available from the statistical center of the State of Delaware (Highway Statistics, [4]) as well as the AVMT from the statistical department of the State of Maryland (‘HISD Reports’ [5]). In both cases, data are taken from the traffic data of the Interstate Highway 95 (I-95) since it is the primary alternative for trips between cities in the NEC.

The series of prices per car journey can be found in Ref. [9]. These give us different estimations of the cost per mile. In addition, the prices of petrol and by-products can be found in Ref. [11]. Such prices allow us to estimate, in another way, the cost per car and coach journey. The prices per coach journey can be found in Ref. [10].

Fourth, statistics concerning the share of each mode of transport in the cost to the consumer can be found in the BLS, Table 1100 [12]. Detailed tables prior to 2013 need to be requested directly from the BLS.

## 2 Experiment design, materials and methods

The methodology, the theoretical models and the methods are in Escañuela Romana, I. (2019) [1]. Some calculations are necessary to be able to finally quantify the number of passengers per each mode of transport and the prices of use that they face each year, especially with the following adjustments.

In relation to car and coach transport, the AVMT series must undergo the following adjustments:

- Be divided by the average distance of the journey per freeway in the NEC.
- Be multiplied by the share of cars and coaches within the total amount of traffic circulation in the US [6].
- Be multiplied by the average number of passengers per vehicle, in cars [7] and in coaches [8].

With regard to the costs per journey by cars and coaches, these must be multiplied by the average distance of the journey in the NEC and divided by the average number of passengers per vehicle [7,8]. However, the data series of prices per coach [10] does not cover the entire time series. This can be extrapolated from the increases expressed in the index numbers of the price series of gasoil [11], with an understanding that both entail a high correlation.

Finally, it is necessary to employ a joint quantification of transport per road: adding the number of passengers and calculating a price weighted by the number of passengers of each mode.

In the series concerning the consumers’ budget share, in Table 1100 [12], we distinguish between the costs per consumer for ‘Gasoline and motor oil’ and ‘Gasoline on out-of-town trips’, and ‘Parking fees’ and ‘Parking fees on out-of-town trips’. Moreover, this Table provides data about the expenditure on: ‘Airline fares’, ‘Intercity bus fares’, ‘Intercity train fares’ and ‘Taxi fares and limousine services on trips’. The expenditures on ‘Vehicle purchases (net outlay)’ and ‘Other vehicles expenses’ do not include this distinction concerning out-of-town trips. In this investigation, I calculated the percentage of expenditures on out-of-town trips in relation to expenditures on the entire related set (e.g. Gasoline and others on out-of-trips as a % of Gasoline and others). I then multiple this value by the different expenditures on cars.

**Fig. 1:**
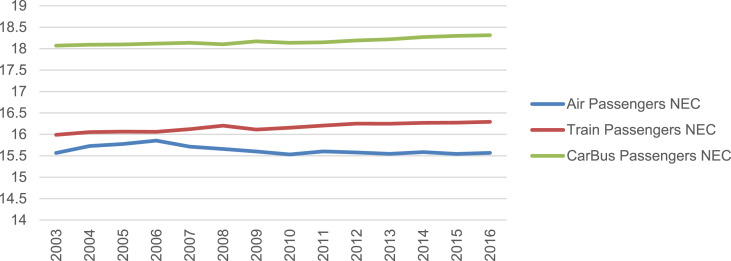
Annual passengers NEC (log).

**Fig. 2:**
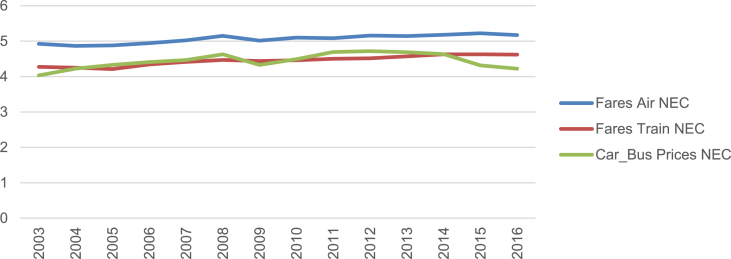
Prices passenger transport NEC (log).

**Fig. 3:**
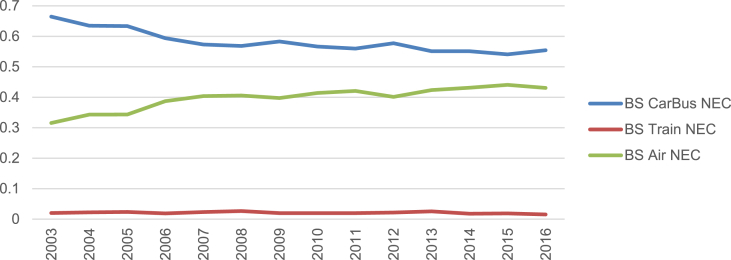
Budget shares.

The final estimation has been obtained by using R programming language (RFoundation for Statistical Computing) and the software package is“systemfit” (Henningsen & Hamann, 2007) [14].Finally, the graphic of the data series in napierian logarithms is the following: (see Fig. 1, Fig. 2, Fig. 3).
